# Organoclay hybrid materials as precursors of porous ZnO/silica-clay heterostructures for photocatalytic applications

**DOI:** 10.3762/bjnano.7.188

**Published:** 2016-12-12

**Authors:** Marwa Akkari, Pilar Aranda, Abdessalem Ben Haj Amara, Eduardo Ruiz-Hitzky

**Affiliations:** 1 Instituto de Ciencia de Materiales de Madrid, CSIC, c/ Sor Juana Inés de la Cruz 3, Cantoblanco, 28049 Madrid, Spain; 2 Laboratory of Physics of Lamellar Materials and Hybrid Nano-Materials (LPLMHNM), Faculty of Sciences of Bizerte, University of Carthage, 7021 Zarzouna, Tunisia

**Keywords:** delamination, montmorillonite, organoclays, photocatalysis, porous clay heterostructures, sepiolite, ZnO nanoparticles

## Abstract

In this study, ZnO/SiO_2_-clay heterostructures were successfully synthesized by a facile two-step process applied to two types of clays: montmorillonite layered silicate and sepiolite microfibrous clay mineral. In the first step, intermediate silica–organoclay hybrid heterostructures were prepared following a colloidal route based on the controlled hydrolysis of tetramethoxysilane in the presence of the starting organoclay. Later on, pre-formed ZnO nanoparticles (NP) dispersed in 2-propanol were incorporated under ultrasound irradiation to the silica–organoclay hybrid heterostructures dispersed in 2-propanol, and finally, the resulting solids were calcinated to eliminate the organic matter and to produce ZnO nanoparticles (NP) homogeneously assembled to the clay–SiO_2_ framework. In the case of montmorillonite the resulting materials were identified as delaminated clays of ZnO/SiO_2_-clay composition, whereas for sepiolite, the resulting heterostructure is constituted by the assembling of ZnO NP to the sepiolite–silica substrate only affecting the external surface of the clay. The structural and morphological features of the prepared heterostructures were characterized by diverse physico-chemical techniques (such as XRD, FTIR, TEM, FE-SEM). The efficiency of these new porous ZnO/SiO_2_-clay heterostructures as potential photocatalysts in the degradation of organic dyes and the removal of pharmaceutical drugs in water solution was tested using methylene blue and ibuprofen compounds, respectively, as model of pollutants.

## Introduction

In the last decades, great effort has been devoted to study ZnO as a very promising catalyst in the photocatalytic degradation of water pollutants. This is because of its elevated activity, its low cost and, in particular, its environmentally friendly behavior [1–2]. It has been conﬁrmed that ZnO compared to TiO_2_ exhibits better efﬁciency in the photocatalytic degradation of organic pollutants [3–6]. It should be remembered that nanoparticulated zinc oxide is a wide-band gap II–VI semiconductor with a band-gap energy of around 3.4 eV, which is of great interest for photocatalytic applications [7]. ZnO nanoparticles (NP) have been assembled to microparticulated layered silicates of the smectite family, giving rise to materials exhibiting interesting properties [8–9]. The immobilization of those NP on clay surfaces represents an advantage for the easier recovering of the photocatalyst from the reaction medium compared to ZnO NP alone.

In recent years, the development of porous heterostructures based on clays attracts many researchers aiming to prepare adsorbents and catalysts for different applications [10]. Amongst the diverse strategies that have been applied in the preparation of these porous materials Letaïef and co-workers [11–12] proposed a methodology in which it was possible to reach the delamination of layered silicates previously exchanged with long-chain alkylammonium cations (organoclays) [13]. In this way, organo-smectites and organo-vermiculites have been used to prepare a new type of nanocomposites consisting of delaminated layered silicates assembled to diverse inorganic NP [10,14]. Moreover, those containing silica-clay entities appear as very attractive materials in view of their elevated specific surface area and the possibility of further functionalization. The more common silica sources used to prepare them are tetraethoxysilane (TEOS) and tetramethoxysilane (TMOS) although this methodology may involve the use of other alkoxysilanes as well as diverse metal alkoxides [14]. The influence of the surfactant incorporated into the organoclay on the characteristics of the obtained materials has been pointed out recently [15–16]. The methodology has been also applied to organoclays derived from fibrous clays (e.g., sepiolite) in which the presence of the surfactant at the external surface results in the formation of silica NP of diverse characteristics depending on the nature of both, silane precursor and surfactant present at the organic–inorganic interface [17]. In general, this sol–gel procedure leads to heterostructured clay-based materials with enhanced textural properties compared to the ones of pristine silicates [14]. The simultaneous use of silicon and metal-alkoxide precursors gives rise, for instance, to the formation of silica/alumina-clay heterostructures providing acid-catalyst behavior [18–19]. Similarly, silica–titania delaminated clays have been also prepared via the generation of TiO_2_/SiO_2_ NP in the interlayer space of smectites and vermiculites modified with long-chain alkylammonium species, promoting a clay delamination that leads to new porous TiO_2_/SiO_2_-layered clay heterostructures [20]. The procedure has been also applied to fibrous clays with the aim to form TiO_2_/SiO_2_ NP anchored on the external surface of sepiolite, leading to porous heterostructures exhibiting photocatalytical properties [21]. In this context, the presence of SiO_2_ NP may play an additional role regarding the improvement of adsorption properties, although this effect has not been clarified neither its possible influence in photocatalytic behavior of resulting materials. Moreover, the simultaneous generation of TiO_2_ and SiO_2_ NP from the two alkoxide precursors may result in the formation of mixed oxides showing poorer photoactivity than the clay heterostructure containing only TiO_2_ NP.

In the present work, we have explored a different approach to prepare ZnO/SiO_2_-clay heterostructures derived from layered silicates and sepiolite fibrous clay, in which SiO_2_-clay organoheterostructures are used for further assembling ZnO NP. In this way, heterostructures in which the surfactant is still present are formed from TMOS and organoclays derived from two types of silicates (smectites and sepiolite) and then the formed hybrid phases are treated with a colloidal suspension of ZnO NP in 2-propanol, following a methodology recently used for the preparation of ZnO/clay nanoarchitectures [22]. The final goal is to reach, in a simple way, ZnO-clay based heterostructures with improved textural properties where ZnO NP remain immobilized and photoactive. To confirm this, the resulting ZnO/silica-clay heterostructured porous solids have been tested in photocatalytic experiments using water solutions of methylene blue (MB) dye or ibupofren drug, as models of organic pollutants, to prove their efficiency as photocatalysts for environmental applications.

## Results and Discussion

### ZnO/silica-montmorillonite heterostructures

**Figure 1:**
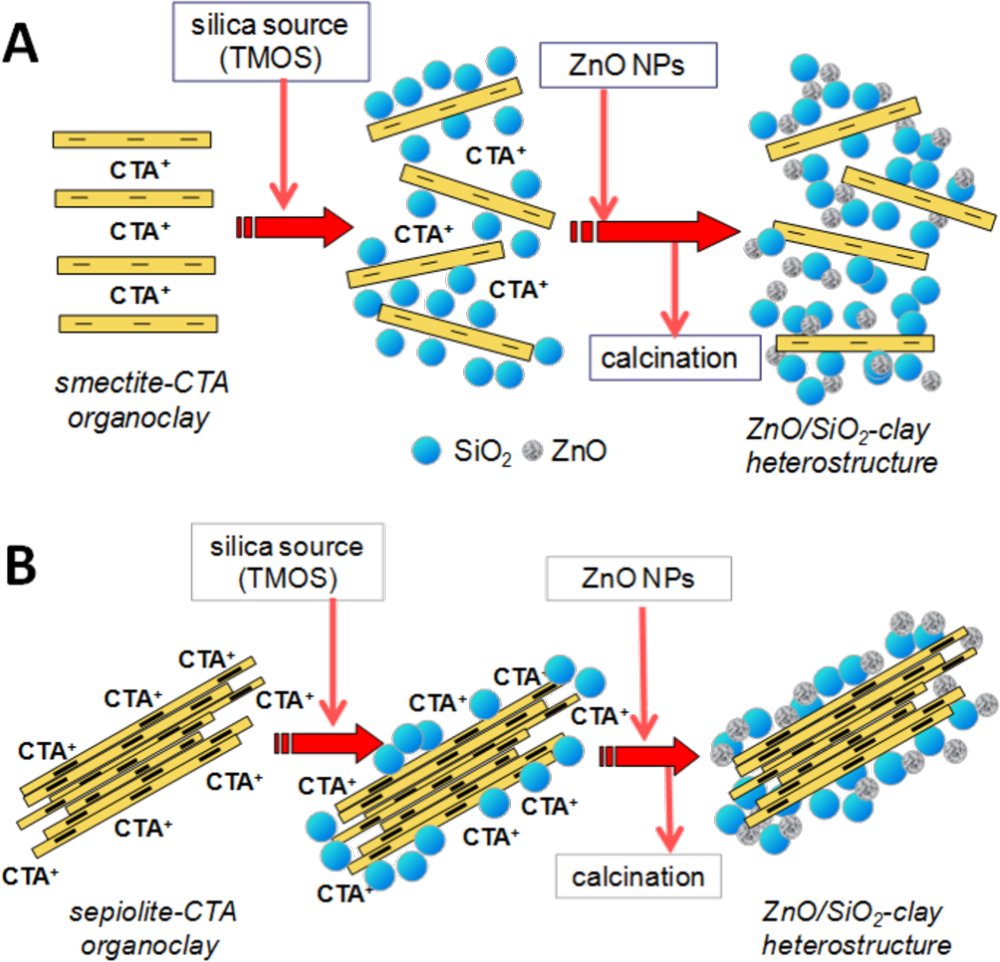
Scheme of the synthetic approach employed in the preparation of the ZnO/SiO_2_-clay heterostructures derived from smectite (A) and sepiolite (B) clay minerals.

The synthesis of ZnO/silica-clay heterostructures was developed following in part the procedure already reported for the preparation of silica-clay nanocomposites [11–12]. The aim is to produce first silica-clay heterostructures in which the growth of SiO_2_ NP from the controlled hydrolysis and polycondensation of TMOS provokes at least a partial delamination of the smectite and so the resulting system will offer a high specific surface area where ZnO NP will be assembled in a second step of the process. As described in the Experimental section, the intermediate/silica-organoclay samples were firstly prepared and, in a second step, were treated with freshly synthesized ZnO NP dispersed in 2-propanol (Figure 1A).

**Figure 2:**
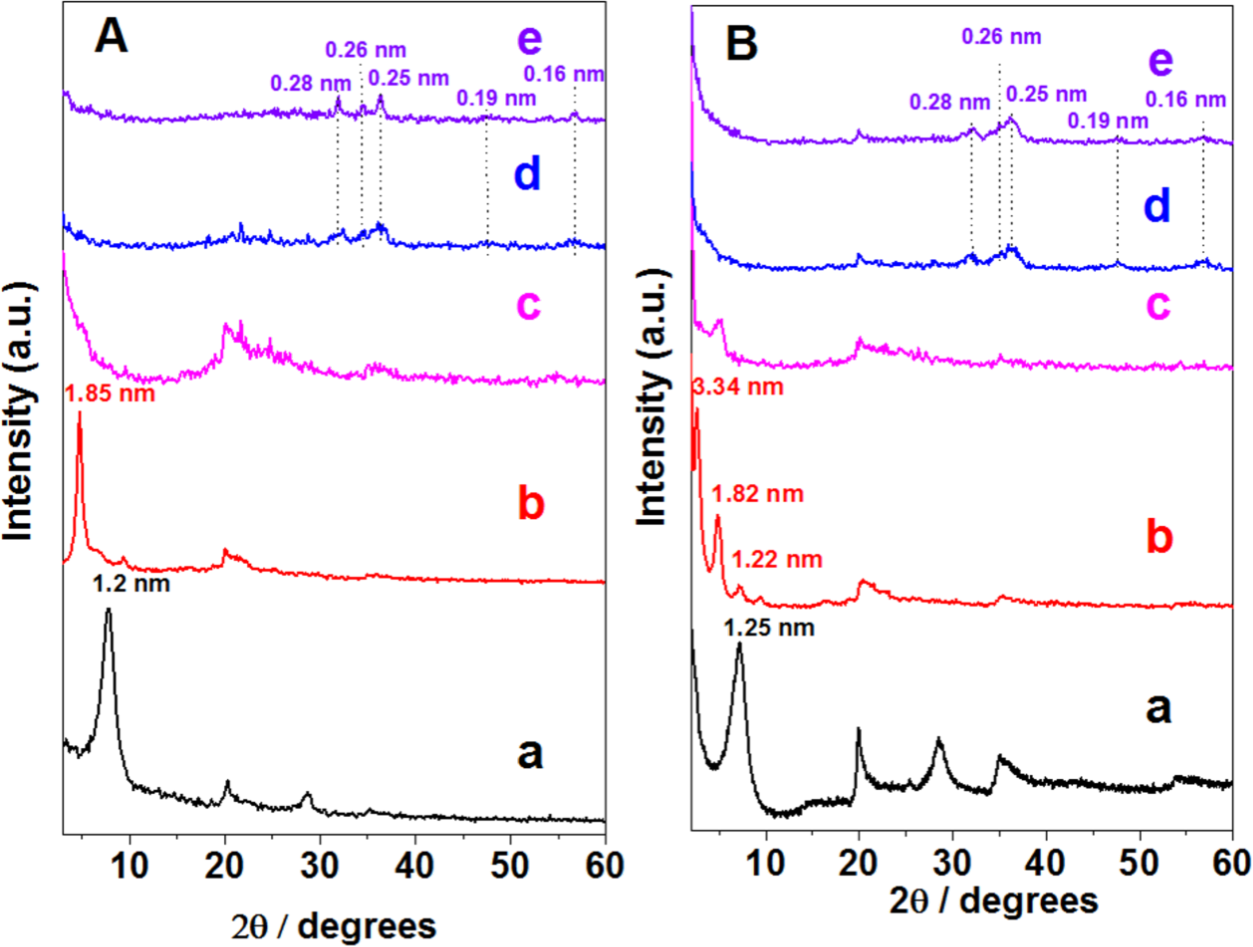
XRD patterns for Cloisite^®^ (A) and TSM (B) corresponding to: (a) the starting clay, (b) CTA^+^-exchanged clays (organoclays), (c) intermediate SiO_2_-organoclay samples, (d) ZnO/SiO_2_-clay heterostructures before calcination, and (e) ZnO/SiO_2_-clay after calcination at 500 °C. Starting clays and organoclays are the same than in the previous study [22].

The XRD diagrams (Figure 2) of the two layered silicates treated with CTAB, Cloisite^®^ (CLO-CTA) and smectite from the Gafsa region (TSM-CTA), indicate an increase of the interlayer spacing (*d*_001_) from ca. 1.2 nm in the pristine Na^+^-exchanged clays to ca. 1.85 nm (Figure 2Ab) and 3.34 nm (Figure 2Bb), respectively. This feature is due to the different degree of ion exchange and the different conformation in which the CTA^+^ surfactant ions are intercalated in each type of layered silicate. After the generation of the SiO_2_ network the XRD diagrams change drastically and the most intense (001) reflection is practically undetectable suggesting a loss of the stacking of the silicate layers through delamination (Figure 2Ac and Figure 2Bc). This feature is maintained in both smectite samples after assembling of the ZnO NP before and after the thermal treatment (i.e., diffractograms d and e in Figure 2A) and it is ascribed to the silicate delamination, which has also been reported in other layered systems [11,19–20]. The only difference between the XRD diagrams of the samples before and after thermal treatment refers to the presence of more intense diffraction peaks at 0.28, 0.26, 0.25, 0.19 and 0.16 nm in the final ZnO/SiO_2_-clay heterostructures, which correspond to the (100), (002), (101), (102) and (110) reflections assigned to ZnO hexagonal wurtzite lattice (JCPDS 36-1451), respectively. The particle size calculated from these peaks using the Debye–Scherrer equation (vide infra) are 20 and 9 nm for ZnO/SiO_2_-CLO and ZnO/SiO_2_-TSM samples, respectively, which is close to the dimensions observed by TEM.

The characteristics of the heterostructures at the different stages of preparation were also evaluated by FTIR (data not shown). In both ZnO/SiO_2_-organoclay heterostructures, the presence of bands at around 2920 and 2850 cm^−1^ as well as in the 1470 cm^−1^ region is clearly observed. These bands are typical for the ν_C–H_ stretching and δ_CH2_ deformation vibration modes of –CH_2_– and CH_3_– groups belonging to the CTA^+^ ions, respectively. After the thermal treatment, these bands completely disappear from the infrared spectrum corroborating the whole removal of the organic species by calcination in accordance also with results from thermogravimetric curves (data not shown). The bands in the 950–1200 cm^−1^ region correspond to the ν_Si–O_ stretching vibration modes of the clay, typically appearing as an intense band centered in the range of 1020–1050 cm^−1^ with a shoulder around 1100 cm^−1^. In the samples containing SiO_2_ the shoulder shifts to higher wavenumbers and even a second shoulder is observable in the 1180–1200 cm^−1^ range, as the bands ascribed to vibration modes of the silica network overlap those of the clay substrate, as it was also observed in other SiO_2_-clay nanocomposites [11].

**Figure 3:**
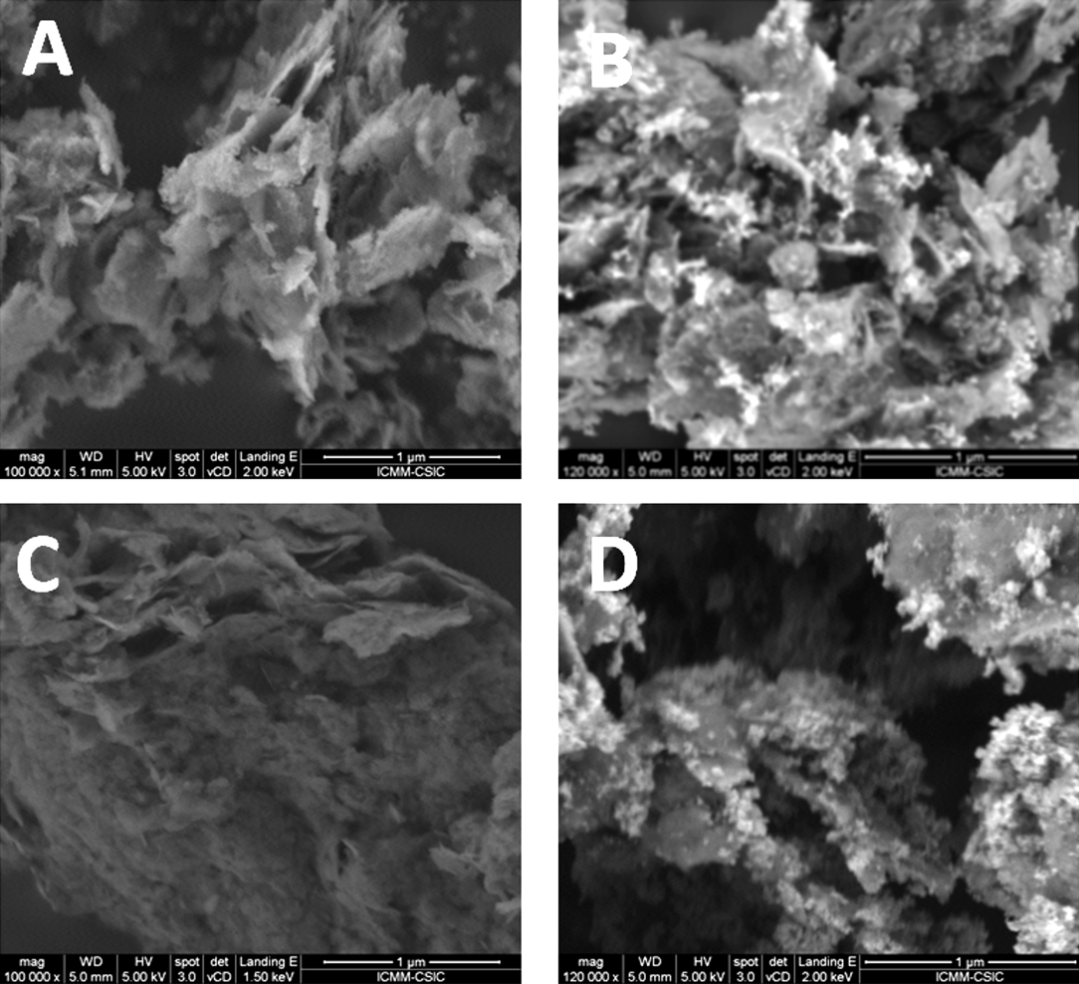
FE-SEM images of the heterostructures: (A) SiO_2_-CLO-CTA, (B) ZnO/SiO_2_-CLO, (C) SiO_2_-TSM-CTA and (D) ZnO/SiO_2_-TSM.

**Figure 4:**
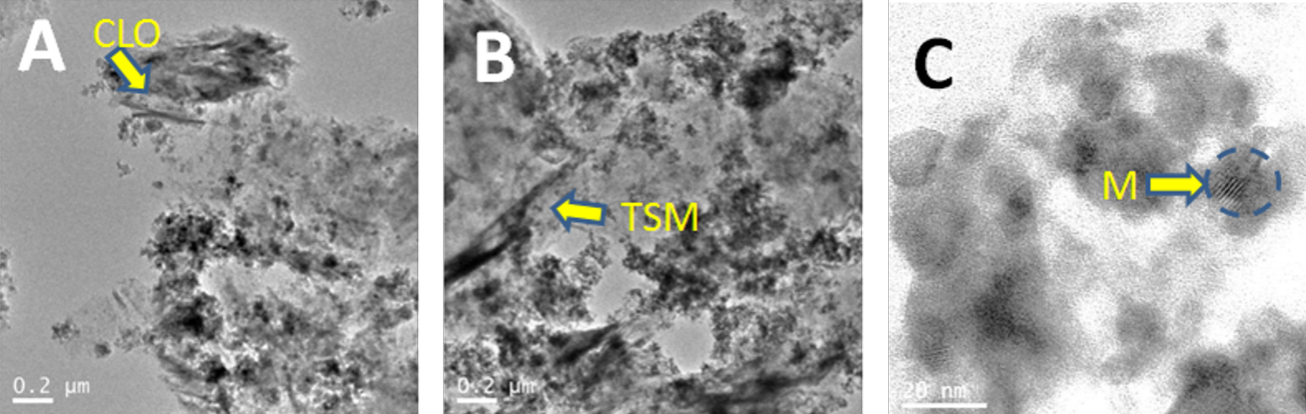
TEM images of the heterostructures: (A) ZnO/SiO_2_-CLO and (B) ZnO/SiO_2_-TSM, in both images the arrows show regions with the presence of delaminated clay particles (CLO and TSM, respectively). Image (C) represents a detail of A at higher magnification showing a ZnO nanoparticle within the silica matrix (M arrow: Moiré fringe).

FE-SEM images (Figure 3) show the typical spongy morphology of SiO_2_-organosmectite materials which is also preserved in the ZnO/SiO_2_-clay heterostructures formed after thermal treatment at 500 °C, detecting in the latest the presence also of ZnO NP (Figure 3B and Figure 3D). From the TEM images corresponding to the final heterostructures (Figure 4) one can observe: i) the presence of delaminated clay platelets (the region indicated by the arrow in Figure 4A and 4B); ii) the presence of ZnO NP (showing in some cases Moiré fringes, Figure 4C); and iii) the presence of aggregates of SiO_2_ NP which in some cases may remain assembled to ZnO particles, perhaps organized even as core–shell structures (Figure 4C). EDX analysis of ZnO/silica-clay heterostructures shows the presence of Zn in a significant amount with respect to the Si content in all the samples. However, it is complicated to estimate the precise ZnO/clay/SiO_2_ ratio because of the difficulty to ascertain how much of the Si contribution is coming from the clay silicate and how much from the generated silica.

**Figure 5:**
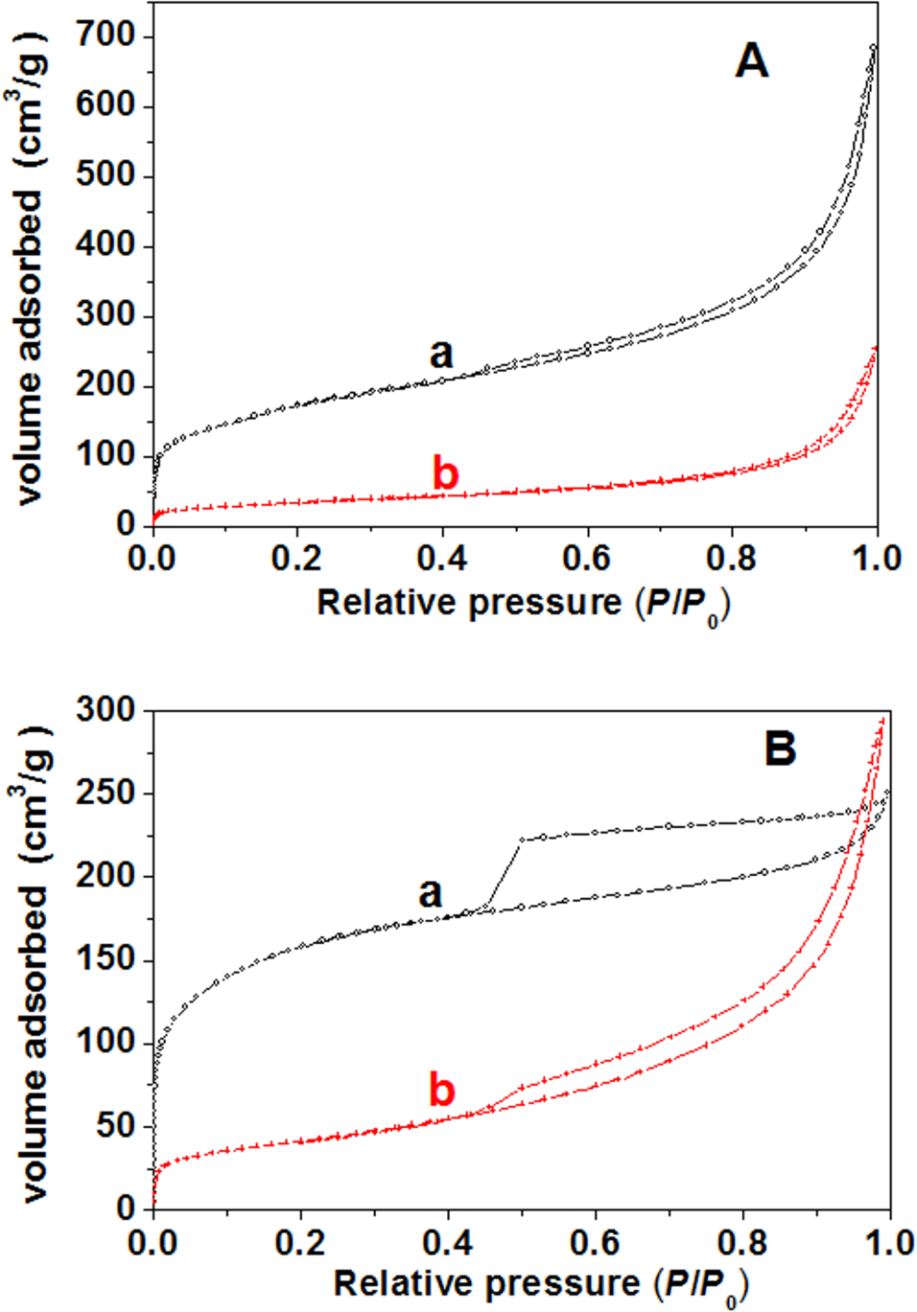
N_2_ adsorption–desorption isotherms (77 K) of SiO_2_-clay (a) and ZnO/SiO_2_-clay (b) heterostructures based on Cloisite^®^ (A) and TSM (B) clays, respectively.

The porous nature of ZnO/SiO_2_-montmorillonite materials was characterized by nitrogen adsorption–desorption isotherms at 77 K (Figure 5), obtained as described in the Experimental section [12]. The ZnO/SiO_2_-montmorillonite heterostructure derived from Closite^®^ shows an type-I/II isotherm with a H3-type hysteresis loop, according to the IUPAC classification [23]. This isotherm is compared in Figure 5A with the one of the SiO_2_-organoclay without assembly of ZnO NP but submitted to a similar thermal treatment. The silica-clay (SiO_2_-CLO) heterostructure is a porous material the isotherm of which exhibits a comparable trend with a greater adsorption capacity but also shows the presence of microporosity. This microporosity in the ZnO/SiO_2_-CLO heterostructure is probably blocked by the ZnO NP. In the case of the ZnO/SiO_2_-clay heterostructure derived from TSM smectite, the isotherm type is also quite similar to that of the ZnO/SiO_2_-CLO heterostructure with scarce microporosity. Clearly, the related SiO_2_-TSM heterostructure exhibits a slightly different type of isotherm (type I with H4 hysteresis loop) (Figure 5B). The textural parameters calculated from these isotherms are summarized in Table 1, where they are compared to related porous materials including the SiO_2_-clay hetererostructures without ZnO NP and previously reported ZnO-clay heterostructures prepared by direct assembly of ZnO NP to the organoclays (without silica incorporation) [22]. The ZnO/SiO_2_-clay samples show specific surface areas of around 126 and 148 m^2^/g for Cloisite^®^ and TSM smectites, respectively. By comparing these values to those of the related ZnO-clay materials [22] a significant increase in the specific surface area values in the heterostructures prepared incorporating previously silica is clearly evident. However, it should be noted that these materials exhibit reduced specific surface area values compared to that developed by silica-clay heterostructures obtained by calcination under the same conditions but without assembling of ZnO NP (Table 1). Anyway, as it occurs with other related materials affected by delamination processes, the solids prepared here exhibit higher values of total porosity and specific surface area than ZnO-clay heterostructures, which is of paramount importance for catalytic purposes.

**Table 1:** Textural characteristics of diverse smectite- and sepiolite-based heterostructures calculated from their N_2_ adsorption–desorption isotherms (77 K).

sample	*S*_BET_^a^ (m^2^/g)	*S*_ext_^b^ (m^2^/g)	V_MP_^c^ (cm^3^/g)	V_T_^d^ (cm^3^/g)

ZnO/SiO_2_-CLO	126	135	—	0.367
SiO_2_-CLO	604	574	0.0155	0.987
ZnO/CLO^e^	95	94	—	0.252
ZnO/SiO_2_-TSM	148	137	0.0459	0.433
SiO_2_-TSM	518	360	0.0857	0.377
ZnO/TSM^e^	51	48	0.0007	0.200
ZnO/SiO_2_-SEP	111	94	0.0089	0.354
SiO_2_-SEP	332	176	0.0822	0.455
ZnO/SEP^e^	104	83	0.0104	0.366

^a^Specific surface area from BET method, ^b^specific external surface area, ^c^micropore volume calculated by the t-method and, ^d^total pore volume at *P*/*P*_0_ = 0.99; ^e^values from reference [22].

### ZnO/SiO_2_-sepiolite heterostructures

The preparation of ZnO/sepiolite heterostructures in which sepiolite was previously modified by assembling of SiO_2_ NP has been also explored with the aim to increase the accessible surface area to ZnO NP. As indicated in the Experimental section, the resulting SiO_2_-organosepiolite material was used in a second step for the assembly of freshly synthetized ZnO NP following a protocol similar than the one applied for the preparation of the smectite-based heterostructures (Figure 1B).

**Figure 6:**
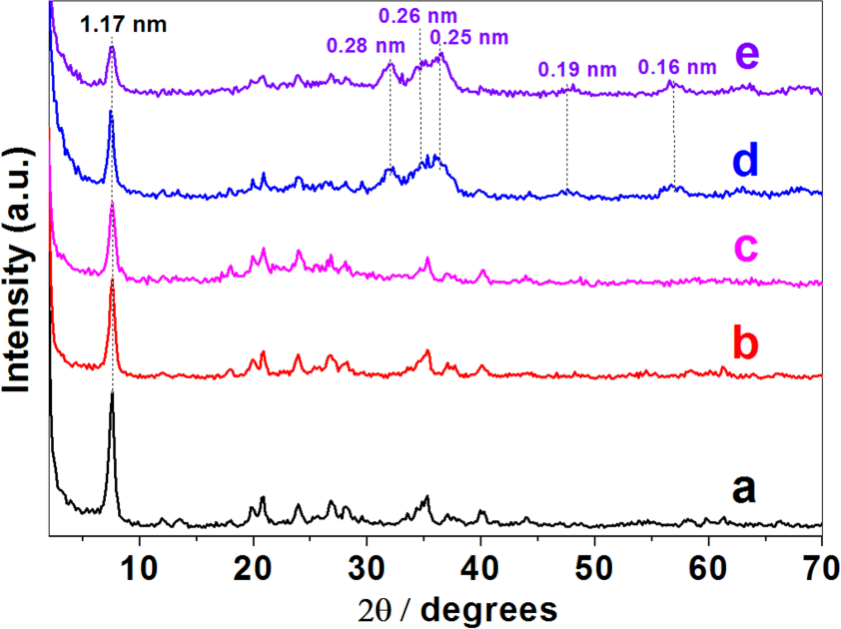
XRD patterns of (a) starting sepiolite (SEP), (b) SEP-CTA organoclay, and (c) SiO_2_/SEP-CTA, (d) ZnO/SiO_2_-SEP-CTA and (e) ZnO/SiO_2_-SEP heterostructures. Samples SEP and SEP-CTA are the same than in the previous study [22].

XRD patterns of pristine sepiolite fibrous clay and the organoclay obtained by treatment with CTAB revealed not changes, confirming that the surfactant is just assembled to the external surface of the clay (Figure 6). This technique also reveals that the hydrolysis and polycondensation of TMOS to produce silica NP on the organo-sepiolite surface did not cause any relevant structural change, as it was already reported for related titania-clay heterostructures [21]. The further assembly of ZnO NP to produce the final ZnO/SiO_2_-SEP heterostructure is confirmed by the presence of new peaks in the corresponding XRD patterns of the heterostructure before and after the calcination step (Figure 6d and Figure 6e). As occurs in the heterostructures derived from layered clays, here the presence of ZnO NP stabilized in the wurtzite phase (JCPDS 36-1451) is also observed. Interestingly, the calcination procedure here applied did not cause the complete structural collapse of the sepiolite, as revealed by the presence of the (110) reflection in all the patterns, being still quite intense and centered at around 1.17 nm in the XRD diagrams of the final heterostructure (Figure 6e). Comparable behavior has been also reported for diverse heterostructured materials based on sepiolite, where organic or inorganic components can partially penetrate the sepiolite structural tunnels impeding the silicate folding [18,24].

**Figure 7:**
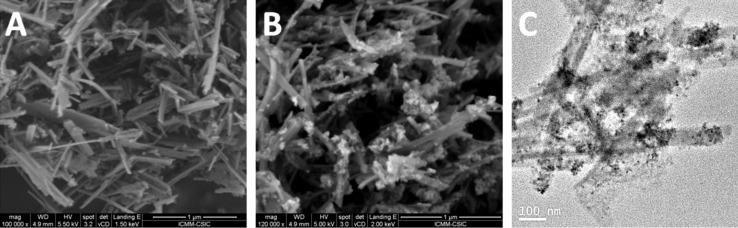
FE-SEM images of SiO_2_-SEP-CTA (A) and ZnO/SiO_2_-SEP (B) heterostructures, and TEM image of ZnO/SiO_2_-SEP heterostructure (C).

**Figure 8:**
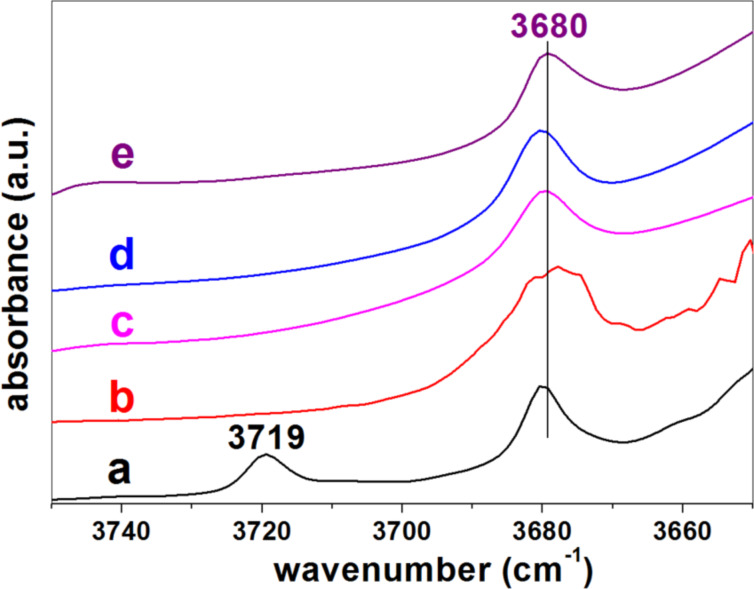
FTIR spectra (3750–3650 cm^−1^ region) of (a) pristine sepiolite (SEP), (b) SEP-CTA, and the (c) SiO_2_/SEP-CTA, (d) ZnO/SiO_2_-SEP-CTA and (e) ZnO/SiO_2_-SEP heterostructures. Samples SEP and SEP-CTA are the same than those in the previous study [22].

From the FE-SEM images of the SiO_2_-SEP-CTA sample before (Figure 7A) and after the assembly of ZnO NP to form the heterostructure ZnO/SiO_2_-SEP (Figure 7B) it is clearly observed the presence of the metal oxide NP associated with the clay. In the SiO_2_-SEP-CTA heterostructure (Figure 7A) the fibers covered by a coating, which after calcination is transformed in more discrete nanoparticles of both SiO_2_ and the assembled ZnO NP, can be seen clearly (Figure 7B). TEM images of the ZnO/SiO_2_-SEP heterostructure (Figure 7C) show more clearly the presence of sepiolite fibers surrounded by the generated SiO_2_ nanoparticles and by ZnO NP showing pseudo-spherical morphology. A good homogeneity in the ZnO NP distribution on the sepiolite surface is corroborated from these images. Furthermore, both types of NP remain attached to the silicate covering almost completely the fibers anchored to the silanol (Si-OH) groups located at the external surface of sepiolite, which can be confirmed by FTIR spectroscopy (Figure 8). Thus, the ν_O–H_ stretching vibration mode of the Si–OH groups appearing around 3720 cm^−1^ in the spectrum of pristine sepiolite is neither detectable in the spectrum of the organo-sepiolite nor in that of the sepiolite heterostructures. The disappearance of this band is attributed to the direct interaction of silanol groups with the diverse species located at the sepiolite surface in each case. Proof of this is the fact that the ν_O–H_ stretching vibration band ascribed to Mg-OH groups appearing at 3680 cm^−1^ remains unaltered in all the spectra, which was expected as they are located at the interior of the structural blocks of sepiolite without access to the adsorbed species on the silicate [18,24].

**Figure 9:**
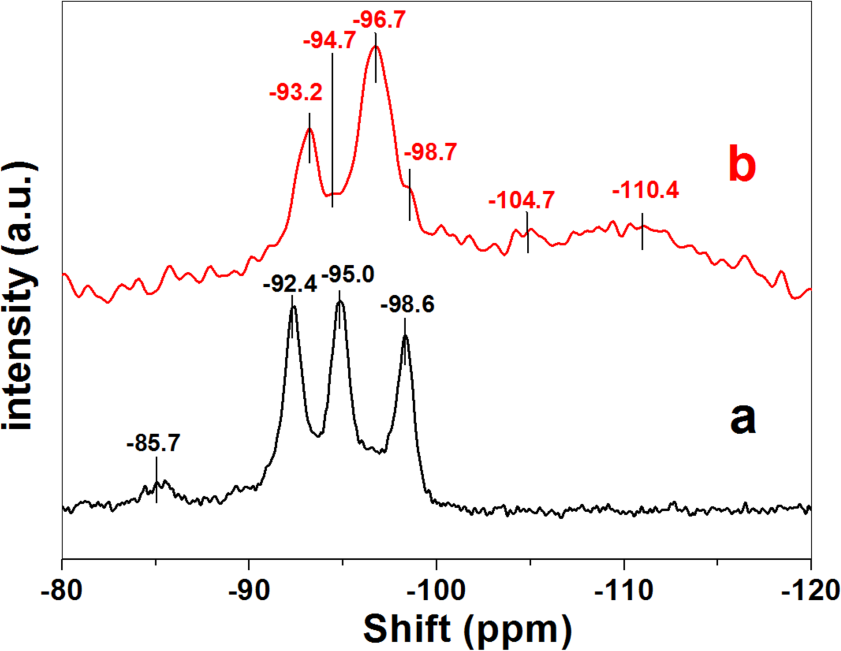
^29^Si solid-state NMR spectra of (a) sepiolite and (b) ZnO/SiO_2_-SEP heterostructure.

Concerning the ^29^Si NMR spectra (Figure 9), the one of the ZnO/SiO_2_-SEP heterostructure is complex as it is composed of ^29^Si signals coming from silicon nuclei of sepiolite structure, from generated silica with different condensation degrees as well as from some other components involving more complex interactions, e.g., silica in interaction with ZnO and sepiolite. ^29^Si NMR spectra of pure sepiolite shows three characteristic signals at approximately −92.4, −95.0, and −98.6 ppm (Figure 9a) typical of Q^3^ signals attributed to Si atoms in different structural environments [25]. There is also a small Q^2^ signal at −85.7 ppm, which is related to the silanol groups located at the surface of the silicate [25]. The ^29^Si NMR spectrum of the ZnO/SiO_2_-SEP heterostructure is clearly different showing a strong decrease in the intensity of the Q^2^ signal, which is attributed to the reaction of sepiolite silanol groups with TMOS. This is similar to reports of other authors related to sepiolite-based heterostructures [17,19,26]. Simultaneously the Q^3^ signals are highly perturbed and the ones of sepiolite are overlapped with those signals from the formed silica, with only two well-defined peaks appearing at −93.2 and −96.7 ppm with two shoulders at −94.7 and −98.7 ppm. In addition, a signal at −104.0 ppm and a large band centered at about −110 ppm are observed. These are related to Q^4^ signals of silica involving Si in different environments and probably also those related to silica–ZnO interactions.

**Figure 10:**
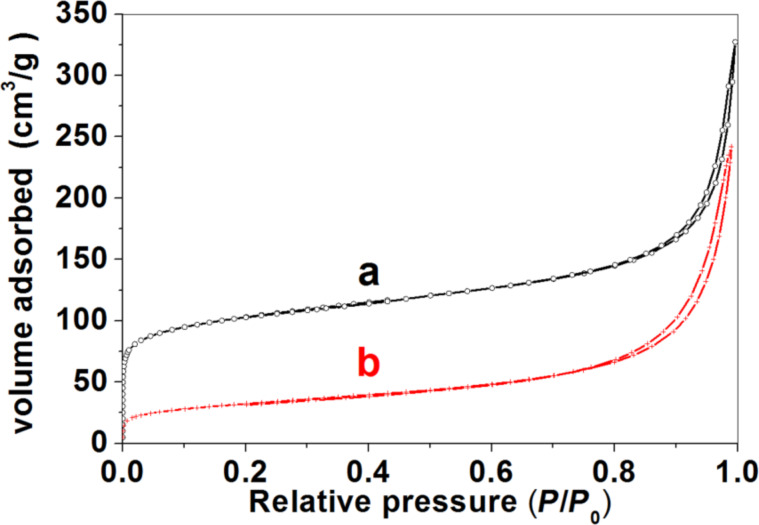
N_2_ adsorption–desorption isotherms at 77 K for SiO_2_-SEP (a) and ZnO/SiO_2_-SEP (b) heterostructures.

The N_2_ adsorption isotherm for the ZnO/SiO_2_-sepiolite heterostructure (Figure 10) is assigned to the type I/II with hysteresis loops of type H3 according to the IUPAC classification. For comparison, a similar isotherm corresponding to the SiO_2_-sepiolite heterostructure prepared from SiO_2_-SEP-CTA by calcination under the same conditions applied to prepare the ZnO/SiO_2_-sepiolite heterostructure is also shown. From Table 1, the significant decrease of the specific surface area from ca. 331 m^2^/g to ca. 111 m^2^/g calculated for silica-sepiolite and ZnO/silica-sepiolite heterostructures, respectively, can be seen. This decrease in surface area can be attributed to the presence of ZnO NP, which partially blocks the tunnels of the sepiolite as well as the porosity created by the SiO_2_ NP attached to sepiolite. A collapse of sepiolite after the thermal treatment at 500 °C (folding of the sepiolite structure) [27] can be ruled out, because XRD revealed not significant structural changes in sepiolite (Figure 6e).

### Catalytic properties of ZnO/SiO_2_-clay heterostructures

**Figure 11:**
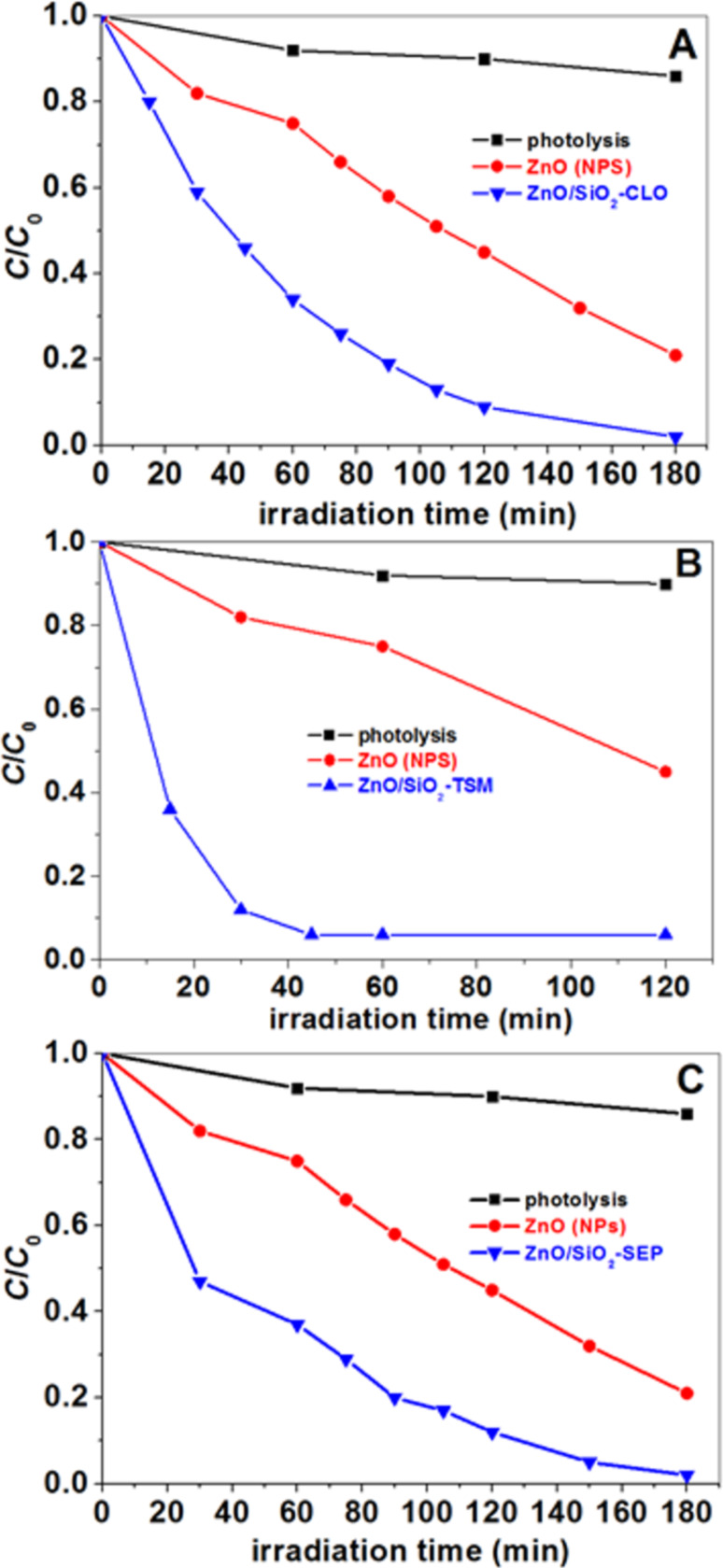
*C*/*C*_o_ (*C*_0_ = 3·10^−5^ M) of MB as a function of the UV irradiation time in presence of the heterostructures (experiments carried out at 17 °C): (A) ZnO/SiO_2_-CLO, (B) ZnO/SiO_2_-TSM and (C) ZnO/SiO_2_-SEP. “Photolysis” corresponds to photodegradation of MB in the absence of catalyst. “Photolysis” and ZnO(NP) data are the same than in the previous study [22].

The ZnO/SiO_2_-clay heterostructures presented here can be of interest for different applications as a photocatalyst. Hence, their activity has been tested using methylene blue (MB) model dye molecule. Figure 11 shows the concentration of methylene blue solutions (*C*/*C*_0_) as a function of the UV irradiation time in presence of SiO_2_/ZnO montmorillonite and ZnO/SiO_2_-sepiolite heterostructures acting as photocatalysts. Apparently, ZnO/SiO_2_-clay materials act as efficient photocatalysts as MB is completely degraded after 180 min of irradiation in presence of ZnO/SiO_2_-SEP or ZnO/SiO_2_-CLO heterostructures, and after only 120 min in presence of the ZnO/SiO_2_-TSM clay heterostructure. It should be noted that the ZnO-heterostructures based on clays are photocatalytically more active when they were modified by introducing silica to improve the textural characteristics. Table 2 shows a comparison of the photoactivity of diverse clay-ZnO systems in the degradation of MB. Probably, the higher activity of ZnO/SiO_2_-clay heterostructures compared to the ZnO-clay materials maybe related to the much higher specific surface area and the larger pore volume of the former. This could also lead to a removal of the pollutant by adsorption instead of just photocatalytic degradation. The current results suggest that an appropriate pore volume with an optimized pore size distribution besides a large specific surface area of these materials, can promote a more efficient photocatalytic activity but also a higher adsorption capacity of MB molecules, which also contributes to the removal of these molecules from solution. This is especially relevant in the present case as MB is a cationic molecule that may be adsorbed by the clay component due to electrostatic interactions in an ion-exchange mechanism. The initial amount of MB adsorbed by the different substrates is in the range of 8–12 mmol/100g. The photoactivity efficiency is determined considering the initial concentration of MB after the adsorption process and measuring its evolution with irradiation time from that point. Moreover, the possibility that MB was only partially degraded reaching structural changes in the molecule that affect the solution color and hence the UV–vis results should be considered. In this sense, work is undergoing to clarify the degradation mechanism when using these porous solids as well as their efficiency of the removal of various pollutants in water.

**Table 2:** The degradation percent of MB aqueous solution over different samples under UV irradiation in comparison with literature.

sample	photodegradation efﬁciency (%) (after 120 min)	reference

ZnO/SiO_2_-SEP	87	this work
ZnO/SiO_2_-CLO	90	this work
ZnO/SiO_2_-TSM	96	this work
ZnO/SEP	84	[22]
ZnO/CLO	96	[22]
ZnO/TSM	62	[22]
ZnO/montmorillonite	40% (150 min)	[28]
ZnO/montmorillonite	15.7 (50 min)	[29]

**Figure 12:**
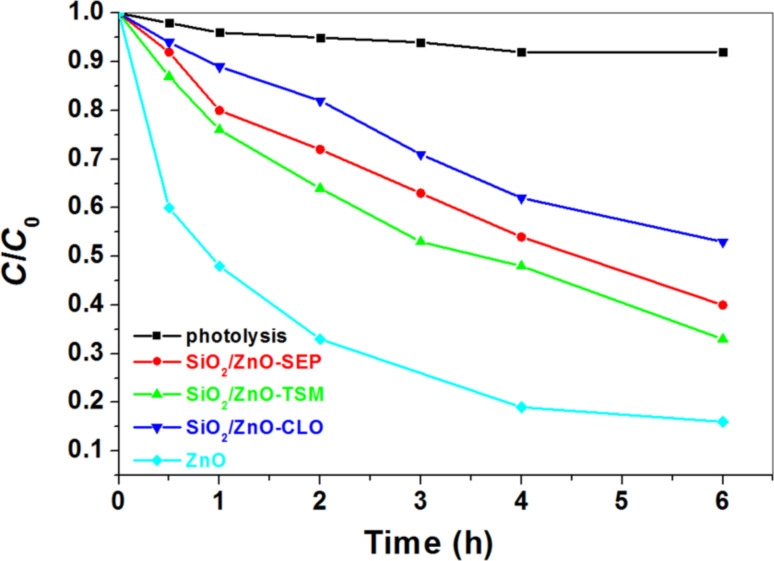
Photoactivity of ZnO NP and ZnO-SiO_2_-clay heterostructures showing degradation of ibuprofen in aqueous solution (*C*_0_ = 15 mg/L) under UV irradiation (experiments carried out at 17 °C).

To explore the potential application of these ZnO-based materials as photocatalyst for the degradation of drug pollutants in water, ibuprofen was selected as a model pharmaceutical. Ibuprofen shows a low adsorption affinity towards silica/silicate substrates and the observed degradation could be directly related to the photocatalytic activity of the tested materials. Figure 12 displays the evolution of the ibuprofen concentration after 6 h of UV irradiation. In absence of catalysts, i.e., the photolysis experiment, there is only 5% of degradation after 360 min, indicating that ibuprofen shows good photostability under UV–vis irradiation. The ZnO/SiO_2_-clay heterostructures clearly show photoactivivity in the degradation of ibuprofen under UV irradiation. The highest activity was observed for the ZnO/SiO_2_-TSM sample (65% degradation after 6 h irradiation), followed by ZnO/SiO_2_-SEP and ZnO/SiO_2_-CLO samples that show 60% and 45% degradation, respectively. Contrarily to that observed in the MB photodegradation study, ZnO nanoparticles alone exhibit a higher activity compared to the activity of the heterostructures, reaching 84% of ibuprofen decomposition after 6 h of irradiation. This feature could be tentatively explained by admitting a more effective UV shielding in the reaction medium under the conditions of ibuprofen study, where the concentration of solids was slightly higher than the one used in the case of MB degradation (see above). It should be also noted that the ibuprofen degradation by the ZnO NP synthesized in this work is lower than that already reported for other ZnO NP, which could be attributed to differences in the NP size [30].

## Conclusion

We introduced a new family of ZnO/SiO_2_ porous clay heterostructures synthesized from organoclay hybrid materials the interfaces of which turned out to be especially useful for the growing and assembling of silica and ZnO NP. The involved clays were layered silicates of the smectite type (a Wyoming montmorillonite commercialized under the name Cloisite^®^ and a Tunisian smectite from the Gafsa region) as well as the sepiolite fibrous clay from Vallecas-Vicálvaro (Spain). Both smectites can be exfoliated during the incorporation of SiO_2_ NP giving rise to delaminated heterostructures, whereas sepiolite is modified exclusively on its external surface. The SiO_2_ NP are generated by controlled hydrolysis and polycondensation of TMOS on the organophilic surface of both types of silicates previously treated with CTAB surfactant to form the corresponding organoclays. The second step in this synthetic strategy consists in the incorporation of ZnO nanoparticles to the SiO_2_-organoclay heterostructures before calcination. As it has been previously observed [22], the presence of the surfactant offers a more convenient interface for the further assembly of ZnO NP. Thermal treatment of these resulting intermediate materials eliminates the organic matter, generating ZnO/SiO_2_-sepiolite and ZnO/SiO_2_-montmorillonite heterostructured materials. In the case of montmorillonite the process is accompanied by the delamination of the involved clay [12,19–20]. Interestingly, the heterostructures described here exhibit advantages, such as a larger specific surface area, over related materials prepared by the direct attachment of metal oxide NP to the clay without incorporation of silica NP [22]. Moreover, the investigation on the photocatalytic ability of the present materials indicates that ZnO/SiO_2_-clay heterostructures derived from smectites showed an enhanced photocatalytic activity for the degradation of methylene blue, probably due to the most suitable textural features among these ZnO-silica clay heterostructures. The observed activity in the degradation of ibuprofen points out to the potential applicability of these catalysts for the removal of pharmaceuticals present in domestic water. The encouraging preliminary results for the obtained catalytic tests should be confirmed in further studies involving various experimental conditions as well as the analysis of the nature of the degraded organic molecules and studies extended to other pollutants.

## Experimental

### Materials

The starting clays used in this work were i) the commercial sodium montmorillonite named as Cloisite^®^-Na (abbreviated as CLO), supplied by Southern Clay Products; ii) the iron-rich smectite from Gafsa (Tunisia) described elsewhere [31], here noted as TSM; and iii) the sepiolite fibrous clay mineral (SEP) from the Vallecas-Vicálvaro clay deposits (Madrid, Spain) provided by TOLSA S.A. with the commercial trade name of Pangel^®^ S9 (rheological grade), which contains more than 95% of pure sepiolite [32]. Methylene blue dye (MB, C_16_H_18_N_3_SCl, RPE) and the ibuprofen sodium salt (C_13_H_17_NaO_2_) were supplied by (Analyticals Carlo Erba) and Sigma-Aldrich, respectively. Hexadecyltrimethylammonium bromide (cetyltrimethylammonium bromide, CTAB, purum, Aldrich) was used in the preparation of the CTA-clay derivatives. Ultra-pure deionized water (18.2 MΩ·cm) was produced in an Elga Maxima Ultra-Pure Water system). Methanol and 2-propanol (Fluka, p.a.) were used as solvents or reactants. Zinc(II) acetate dihydrate (CH_3_COO)_2_Zn·2H_2_O and KOH were purchased from Merck. Tetramethoxyorthosilicate (TMOS) from Fluka (>98 %) was used here as silica precursor.

### Sample preparations

ZnO (NP) synthesis was achieved as reported by Akkari et al. [22]. In a first step, SiO_2_-clay organo-heterostructures (Figure 1) were synthesized by a heterocoagulation reaction involving a sol–gel process with the controlled hydrolysis and polycondensation of silica from silicon-alkoxides in the presence of swollen organosmectites, using the method reported by Letaiëf and co-workers [11–12]. In the present case, the organosmectites were dispersed in 2-propanol and then TMOS was added as silica source to produce materials with a final ratio of 1:1 (SiO_2_/clay). Upon addition of the stoichiometric amounts of water and alcohol (4:2:1 H_2_O/alcohol/TMOS and 1 drop of 1 M HCl) the controlled hydrolysis and polycondensation of the alkoxide took place. The preparation of the SiO_2_-organosepiolite derivative was based on a methodology adapted from a previous one reported by Aranda and co-workers [21]. Shortly, SiO_2_-organosepiolite heterostructure was prepared from a homogenous suspension (5% w/w) of the SEP-CTA organo-sepiolite in 2-propanol to which TMOS was added to produce materials with a final ratio of 1:1 (SiO_2_/clay). This suspension was stirred at 50 °C to homogenize the system and then stoichiometric amounts of water and isopropanol (H_2_O/isopropanol/TMOS 4:2:1 molar ratio) is added to start the hydrolysis of TMOS. After the sol–gel transition the system was dried overnight at 50 °C. The final ZnO/SiO_2_-clay heterostructures were then prepared from this SiO_2_-organoclay dispersed in 2-propanol (5% w/w), to which are slowly added ZnO NP also dispersed in 2-propanol to reach a molar ratio of 0.5:1 ZnO/SiO_2_-organoclay. This suspension is homogenized by ultrasound irradiation (SONICS Vibracell 750 W, 20 kHz) for 20 min in sequential pulses of 10 s of active vibration and 10 s of time-out, using a 13 mm tip operated at 50% amplitude. The resulting solids were dried overnight at 60 °C generating the ZnO/SiO_2_-organoclay samples, which were finally heated to 500 °C for 2 h in N_2_ and 4 h in air, in order to get the desired ZnO/SiO_2_-clay heterostructures. The resulting samples were denoted as ZnO/SiO_2_-CLO, ZnO/SiO_2_-TSM and ZnO/SiO_2_–SEP for the Cloisite^®^, Tunisian clay and sepiolite based materials respectively.

### Characterization of the solids

Powder X-ray diffraction (XRD) diagrams were obtained on a Bruker D8 ADVANCE diffractometer using monochromatic Cu Kα radiation, scanning from 2 to 70° in 2θ degrees with steps of 0.02 degrees. The average size of the crystallites was calculated by using the Debye–Scherrer formula [33]. Fourier transform infrared spectroscopy (FTIR) was performed in a Bruker IFS 260 66v/S spectrophotometer. The samples were prepared as pellets in KBr, in the case of smectite-based materials, or as pure products, in the case of sepiolite-based materials, and the spectra were recorded in the 4000–250 cm^−1^ wavenumber range with 2 cm^−1^ resolution. Transmission electron microscopy (TEM) images were taken using a JEOL 2100F STEM microscopy, operating at 200 kV equipped with an EDX (INCA x-sight of Oxford Instruments) detector for semiquantitative analysis. The specimens for TEM were prepared by putting the as-grown products in ethanol and immersing them in an ultrasonic bath for 15 min, then dropping a few drops of the resulting suspension containing the synthesized materials onto the TEM grid. The morphology of the samples was examined by field-emission scanning electron microscopy (FE-SEM), using a FEI microscope NOVA NanoSEM 230 model coupled to an EDAX Apollo SDD microanalysis system. For visualization, the particle samples were adhered on a carbon tap for direct observation without applying any conductive coating on their surface. Solid-state ^29^Si MAS-NMR spectra were collected on a Bruker Avance 400 spectrometer operating at 79.49 MHz in samples spun at 10 kHz, using a single-pulse sequence of 4.5 and 6 µs recycle delay between accumulations and nearly 3000 accumulations. The ^29^Si chemical shifts were evaluated in relation to tetramethylsilane.

Nitrogen adsorption/desorption isotherms at −196 °C were obtained in a Micromeritics ASAP 2010 analyzer. Before measurement, the samples (150–200 mg) were outgassed under dynamic vacuum for 12 h at 120 °C. The BET specific surface area was calculated from the nitrogen adsorption data in the relative pressure range of 0.05 to 0.2. The external surface area and micropore volume values were achieved by means of the t-plot method according to De Boer’s procedure [34], and the pore volume (*V*_p_) was evaluated from the volume adsorbed at *P*/*P*_0_ = 0.99 in the desorption branch of the corresponding isotherm.

### Photocatalytic activity

Photocatalytic activity of the prepared ZnO/SiO_2_-clay heterostructures was evaluated from degradation of MB and ibuprofen in water, using custom-made equipment. A 300 W UV lamp (Osram Ultra Vitalux E27, 240 V, 300 W, UVA/UVB) with the strongest emission at 354 nm was used as light source and placed above a glass reactor at a distance of 10 cm. Water was circulated through the reactor jacket to ensure a constant temperature of 17 ± 0.5 °C inside the reactor controlled by a thermostatic bath. This temperature below room temperature was chosen to minimize water evaporation.

Batch tests were performed using aqueous solutions of MB and ibuprofen. To a volume of 100 mL containing a 3·10^−5^ mol·L^−1^ MB water solution, were added 20 mg of the ZnO/SiO_2_-clay heterostructures photocatalyst, the mixture being stirred in dark for 30 min to allow for the physical absorption of dye molecules on the catalyst particles to reach equilibrium. In the case of ibuprofen, 25 mg photocatalyst were added to 100 mL of a water solution containing 15 mg·L^−1^ of ibuprofen, the mixture being stirred in dark overnight. Subsequently, the mixture was poured into the glass reactor to start the photocatalytic degradation tests. In these experiments, the reaction solution under magnetic stirring was placed under the UV lamp. The photocatalytic activity of the prepared photocatalyst was compared to that of synthetic ZnO NP under the same conditions. The concentration of MB was determined by measuring the absorption intensity at the maximum absorbance wavelength of MB (663 nm) and ibuprofen (222 nm) by using a UV–vis 2401 PC Shimadzu spectrophotometer. The percentage of the dye degradation was defined as: degradation (%) = (*C*_0_ − *C*)/*C*_0_ × 100, where *C*_0_ is the initial concentration of MB and *C* is the residual concentration of MB at varying intervals of UV irradiation [35].
